# A Rare Presentation of Follicular Odontogenic Keratocyst of Maxillary Sinus Mimicking Dentigerous Cyst: A Case Report with 3-Year- Follow-Up

**DOI:** 10.30476/dentjods.2025.107603.2756

**Published:** 2026-06-01

**Authors:** Satheesh Chandran, Deepak Abraham Pandyan, Deenadayalan Narasimman, Karthik Kattur Premkumar, George Samyo Stephenson, Balamurugan Rajendran

**Affiliations:** 1 Dept. of Oral and Maxillofacial Surgery, Madha Dental College and Hospital, Chennai, India.; 2 Oral and Maxillofacial Surgeon and Oral Implantologist, Dept. of Oral and Maxillofacial Surgery, Rya Madras Cosmo Foundation Hospital, Chennai, India.

**Keywords:** Impacted third molar, Maxillary sinus, Follicular keratocyst, Dentigerous cyst

## Abstract

Follicular keratocyst is a rare variant of odontogenic keratocyst (OKC), which surrounds the neck of an impacted tooth resembling a dentigerous cyst, usually diagnosed through radiographic and histological investigations. A 22-year-old female patient presented with pain in the right maxilla for the past 3 years. Intraoral examination revealed a missing right maxillary third molar. Computed tomography (CT) showed a well-defined hyperdense area in the right maxillary sinus involving the crown of an impacted right maxillary third molar within the cystic lesion. A provisional diagnosis of dentigerous cyst was made. The cystic lesion was then surgically enucleated with an impacted right maxillary third molar under general anesthesia. Histological investigation of the enucleated lesion was suggestive of a follicular keratocyst. No adverse effects or recurrence were elicited postoperatively at the 3-year follow-up. OKC, in relation to the third molar, clinically may represent any other odontogenic cysts. Histopathological examination remains the gold standard for assessing the final diagnosis and guides the selection of a definitive treatment modality.

## Introduction

Odontogenic keratocyst (OKC) is an aggressive developmental cyst that was first described by Philipsen [ 1 - 2 ] in 1956. OKC is a lesion of the jaw that has a propensity for rapid growth and a high recurrence rate, ranging from 0% to 100% [ 3 ]. It generally exhibits a bimodal age distribution, with a first spike in the second and third decades and a second spike in the fifth decade and beyond, showing a male predilection. The most predominant site of OKC occurrence is the mandible [ 4 ], while maxillary third molar and maxillary antrum involvement has been reported to be a rare instance [ 5 - 6 ].

Follicular type OKC accounts for 25-40% of other OKCs, which surrounds the crown and attaches at the neck of an unerupted tooth, mimicking a dentigerous cyst [ 7 ]. Follicular keratocyst presents with pain, swelling, and at times may be asymptomatic. Radiographically, it demonstrates a minimal or extensive involvement of bone surrounding the crown of an impacted tooth. Although the clinical and radiographic interpretations are similar to those of other cystic lesions, the final diagnosis of its variants is determined through histopathological investigation. An appreciable number of OKC have been reported in the literature involving the crown of the third molar [ 8 ]. It was presumed that the dental lamina remnants and the distal offshoots may be accountable for the occurrence of OKC in the third molar [ 9 ], and an alternate theory substantiates the existence of a submucosal hamartoma in OKC situated in the retro-molar area of the mandible and maxillary tuberosity [ 10 ]. This case report illustrates a rare occurrence of a follicular OKC with an impacted third molar in the right maxillary sinus, mimicking a dentigerous cyst. 

## Case Presentation

**Figure 1:**
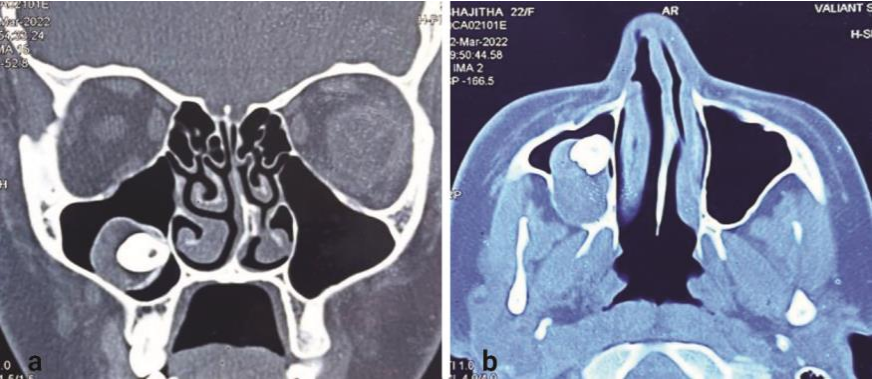
Preoperative computed tomography (CT) showing radiolucency in the right maxillary sinus with presence of impacted right maxillary third molar, **a:** Coronal section, **b:** Axial section

This case report presents a 22-year-old female patient who presented with a complaint of pain in the right maxillary region for the past 3 years. The patient’s history revealed that she had difficulty in breathing, and hence, she was advised to visit an ENT surgeon. She was prescribed with medications, but she could not elicit any positive signs of relief, and the symptoms lasted for a longer duration of time. No gross facial asymmetry was seen on the extraoral examination. Intraorally, there were no signs of tooth eruption pertaining to the maxillary right third molar. Computed tomography (CT) revealed a well-defined hyperdense area in the right posterior alveolar process of the maxilla extending superiorly along the floor of the right maxillary sinus involving the crown of the impacted right maxillary third molar within the lesion. Deviation of the nasal septum to the left side with a septal spur indenting the left inferior turbinate was seen (Figure 1). Based on the clinical and radiographic correlations, the cystic lesion was provisionally diagnosed as a dentigerous cyst. The management of a lesion with an impacted third molar in the right maxillary sinus was planned for surgical enucleation under general anesthesia. 

### Surgical procedure

**Figure 2:**
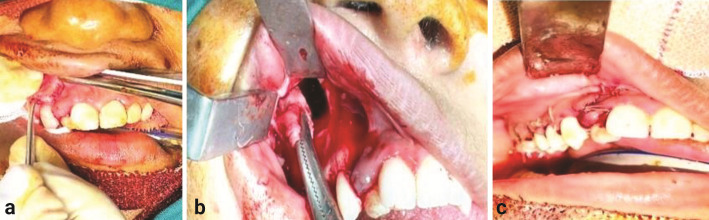
Intraoperative surgical steps, **a:** Surgical incision placed from right maxillary central incisor along the right maxillary vestibule extending up to maxillary third molar region, **b:** A bony window was created and the entire cystic cavity along with the cystic lining and impacted maxillary right third molar was enucleated through Caldwell-Luc approach, **c:** The flaps were approximated with 3-0 vicrylresorbable sutures

**Figure 3:**
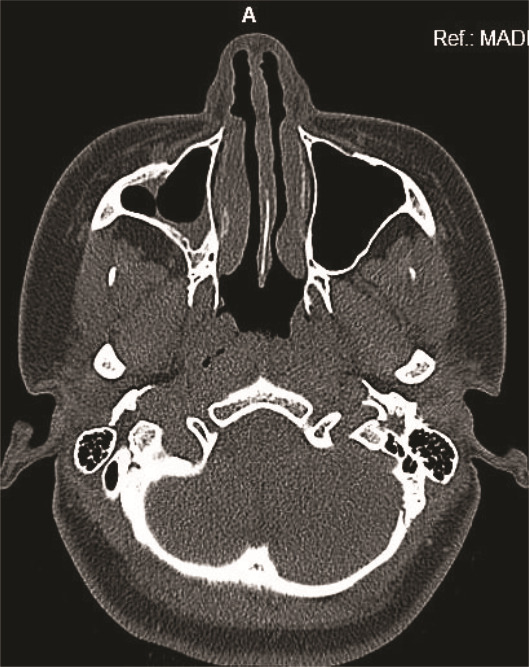
Postoperative computed tomography (CT) showing complete enucleation of the cyst and cystic contents

Under oro-endotracheal intubation, general anesthesia was administered. Standard painting and draping were done using povidone-iodine solution. Local anesthesia was infiltrated (2% lidocaine hydrochloride and 1-in-80,000 concentration of adrenaline) along the right maxillary vestibule. Surgical incision was placed from the right maxillary central incisor along the right maxillary vestibule, extending up to the maxillary third molar region. Mucoperiosteum was then elevated, and a bony window was created in the maxillary first and second molar region extending medially to access the medial wall of the maxillary sinus. The entire cystic cavity, along with the cystic lining and impacted maxillary right third molar, was enucleated through the Caldwell-Luc approach (Figures 2-3). 

Surgical curettage and irrigation with povidone-iodine and normal saline were performed. An absorbable gelatin sponge was placed as a hemostatic agent to prevent hematoma formation. The flaps were then approximated with 3-0 vicryl resorbable sutures. Postoperative instructions were given, and medications (Cefixime tablet 200mg and ketorolac tablet 10mg twice daily) were prescribed for a course of three days. The enucleated specimen was then sent for histopathological examination. 

### Histopathological findings

The soft tissue smear stained with hematoxylin and eosin (H&E) revealed a parakeratinized stratified squamous epithelium with a connective tissue wall of 6-8 cell layer thickness exhibiting palisading cuboidal basal cells, parabasal polygonal cells, and superficial corrugated parakeratin. Several areas of basal cells exhibited hyperplasia and budding, along with occasional entrapment of the epithelial islands within the connective tissue wall. In limited areas, the epithelium appeared to be stripped off or detached from the connective tissue wall. Certain areas showed a thin, non-keratinized epithelial lining that mimicked reduced enamel epithelium, characterized by cuboidal basal and superficially flattened cells of 2-3-layer thickness, which appeared dyscohesive due to underlying inflammation.

The connective tissue wall was collagenous, with several areas of basophilic myxoid degeneration seen along with chronic inflammatory cell infiltrate, moderate vascularity, and areas of hemorrhage. Large and small foci of basophilic cells were observed, and linear and globular dystrophic calcifications were noted within the connective tissue wall. Numerous areas of hyperplastic sinus lining showed pseudo stratified/ ciliated columnar epithelium with goblet cells, and an underlying connective tissue stroma comprising seromucous glands and ducts, with a chronic inflammatory cell infiltrate. 

**Figure 4:**
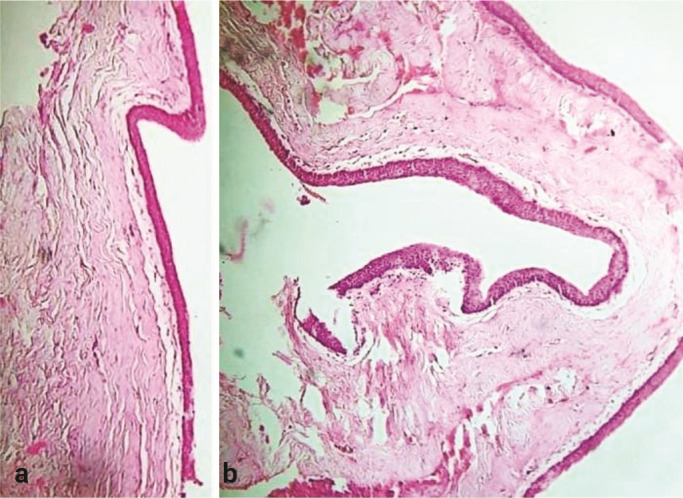
Histopathological examination, **a:** Parakeratinized stratified squamous epithelium with connective tissue wall of 6-8 cell layer thickness exhibiting palisading cuboidal basal cells, parabasal polygonal cells and superficial corrugated parakeratin, **b:** Mature compact and cancellous lamellar bone trabeculae with osteocytes, cemental lines and reversal lines with ragged irregular borders. Fragments of tissues showed a pseudo stratified ciliated columnar epithelium along with underlying edematous connective tissue stroma comprising clumps of chronic inflammatory cells and vascular spaces with moderate vascularity

The hard tissue smear stained with H&E revealed a mature, compact, and cancellous lamellar bone trabeculae with osteocytes, cemental lines, and reversal lines with ragged, irregular borders. Varying amounts of intervening delicate connective tissue stroma were seen along with moderate chronic inflammatory cells, numerous engorged blood vessels, with several areas of hemorrhage. Fragments of tissue showed a pseudo stratified ciliated columnar epithelium, along with an underlying edematous connective tissue stroma comprising clumps of chronic inflammatory cells and vascular spaces. Both soft and hard tissue specimens investigated histologically were suggestive of a follicular keratocyst (Figure 4). 

**Figure 5:**
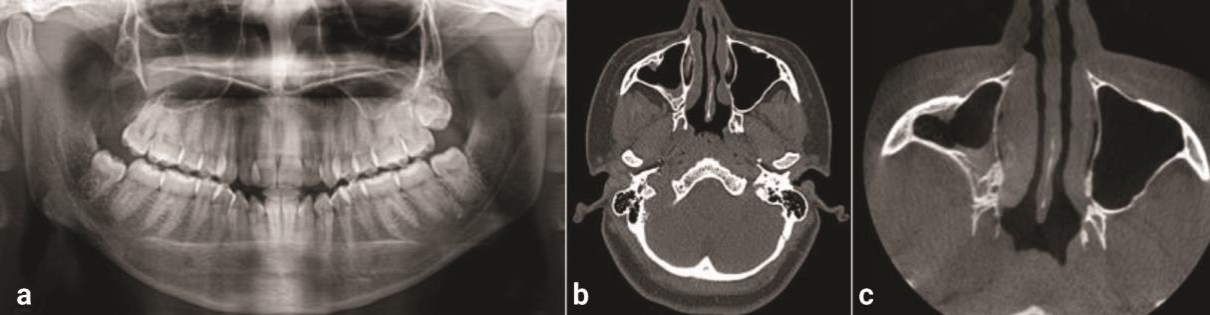
Postoperative follow up images showing no recurrence, **a:** Orthopantamograph (1^st^ year), **b:** Axial section computed tomography (CT) (2^nd^ year), **c:** Axial section computed tomography (CT) (3^rd^ year)

The patient was evaluated for any postoperative adverse effects or complications over a three-year period. No evidence of pain, swelling, discharge, nasal congestion, or any adverse effects was elicited from the patient during the 6-month follow-up period, and there was no recurrence at the 3-year follow-up (Figure 5). 

## Discussion

OKC is a non-inflammatory, developmental cyst of odontogenic origin first coined by Philipsen [ 1 - 2 ] in 1956. Owing to its nature of aggressiveness, OKC was categorized as a neoplasm in 2005, and later it was recognized as a cyst in 2017 [ 11 ]. The follicular variant of OKC is rare, however; only a few cases of follicular OKC have been studied in the literature [ 7 , 12 ]. The term follicular keratocyst was first described by Altini and Cohen [ 12 ] in 1982. It is more commonly seen in females, with peak occurrence in the second and third decades of life. The follicular keratocyst is predominant in the mandible compared to the maxilla [ 4 ]. In contrast, a follicular keratocyst was found in the maxillary area in the current case report.

Clinically, a follicular keratocyst presents with pain, swelling, bone involvement, purulent discharge, and gross facial symmetry. Altini and Cohen *et al*. [ 12 ] described that the cystic lining of a group of lesions showed a significant feature of OKC on histological examination. However, the gross examination showed a cystic lining encircling the crown and attached at the neck of the unerupted tooth. A keratocyst during the phase of enlargement encircles the unerupted tooth follicle, causing fusion of the cystic lining with the reduced enamel epithelium. The cystic epithelium immediately surrounds the neck of the non-keratinized tooth, which in turn exhibits inflammatory changes within the capsule.

Radiographically, a well-defined radiolucency encirrcling the crown of an impacted tooth and the attachment of cystic lining at the neck of the tooth determines a probable differential diagnosis of dentigerous cyst and follicular keratocyst. Histologically, a 6-8 cell layer thickness exhibited palisading cuboidal basal cells, parabasal polygonal cells, and superficial corrugated parakeratin. Certain areas exhibited a 2-3-layer thickness of non-keratinized epithelial lining, resembling reduced enamel epithelium, with cuboidal basal and superficially flattened cells, suggestive of a follicular keratocyst. An immunohistochemical study by Kim *et al*. [ 13 ] stated that the intensity and staining pattern for ki-67 showed positive for both follicular and extra-follicular variants of OKC. Since, follicular OKC and extra-follicular OKC show similarity in their nature of aggressiveness, the treatment must be critically planned to avoid recurrence.

Numerous management protocols [ 7 , 14 ] for follicular keratocysts have been delineated in the literature, which includes decompression, marsupialization, enucleation, and surgical resection based on the size and extent of the cystic lesion. Adjuvant treatment modalities include peripheral ostectomy, electrocauterization, cryosurgery, and chemical cauterization with Carnoy’s solution have proven to show promising outcomes by reducing the recurrence rates [ 14 ]. In our case, the cystic lesion was present within the maxillary sinus. Hence, enucleation of the cyst was performed through the Caldwell-Luc approach, which was in accordance with Madhireddy *et al*. [ 15 ]. Benitha *et al*. [ 7 ] and Spoorti *et al*. [ 16 ] performed enucleation of a follicular keratocyst with peripheral ostectomy in the mandible. 

In the current case report, the patient was evaluated postoperatively for up to three years. No evidence of pain, swelling, discharge, nasal congestion, or any adverse effects was elicited from the patient during the six-month follow-up period, with no signs of recurrence at the three-year follow-up. Benitha *et al*. [ 7 ] and Spoorti *et al*. [ 16 ] showed no recurrence when reviewed at one year. Madhireddy *et al*. [ 15 ] stated that long-term postoperative follow-up is required to assess the rate of recurrence. Moreover, Stoelinga PJW [ 17 ] suggested that lifelong follow-up protocol must be implemented by reviewing patients every year for the first five years and then after every two years.

Follicular keratocyst occasionally portrays the appearances of other odontogenic cysts pertaining to unerupted or impacted teeth. In general, any radiolucent lesion that envelops the impacted tooth’s crown is predominantly diagnosed as a dentigerous cyst, which was not atypical in the present case scenario. Through histopathological investigation, the presence of keratinized lining epithelium significantly demarcates the follicular keratocyst from the dentigerous cyst. This case report clearly defined the importance of histological examination in arriving at a confirmatory diagnosis (Table 1). The limitations of the present case report were that the fine-needle aspiration of the cystic lesion was not performed, which if carried out, would have resulted in a more accurate assessment of the lesion. A long-term follow-up of more than 5 years is required to analyze the recurrence feature of the lesion. 

## Conclusion

Follicular keratocyst denotes a rare phenomenon, particularly enveloping an unerupted tooth’s crown in the maxillary sinus, which masquerades as other cysts of odontogenic origin. A thorough clinical, radiographic, and histopathological assessment, along with molecular perspectives, would guide a more informed closure of the diagnostic perspective and approach to a definitive treatment plan. 
